# Supplementary material for: The Interaction between Intratumoral Microbiome and Immunity Is Related to the Prognosis of Ovarian Cancer
Source: Microbiol Spectr. 2023 Mar 28;11(2):e03549-22. doi: 10.1128/spectrum.03549-22 (PMC10100779; doi:10.1128/spectrum.03549-22)
Supplement: Supplemental file 2 — Supplemental material. Download spectrum.03549-22-s0002.docx, DOCX file, 0.02 MB [file spectrum.03549-22-s0002.docx]

Table S5. Premers for qPCR.

| Gene name | Forward primer | Reverse primer |
| --- | --- | --- |
| TNFA | CCCTCACACTCAGATCATCTTCT | GCTACGACGTGGGCTACAG |
| iNOS | GTTCTCAGCCCAACAATACAAGA | GTGGACGGGTCGATGTCAC |
| ACTB | GGCTGTATTCCCCTCCATCG | CCAGTTGGTAACAATGCCATGT |
