# Supplementary material for: The Interaction between Intratumoral Microbiome and Immunity Is Related to the Prognosis of Ovarian Cancer
Source: Microbiol Spectr. 2023 Mar 28;11(2):e03549-22. doi: 10.1128/spectrum.03549-22 (PMC10100779; doi:10.1128/spectrum.03549-22)
Supplement: Supplemental file 3 — Supplemental material. Download spectrum.03549-22-s0003.pdf, PDF file, 4.2 MB [file spectrum.03549-22-s0003.pdf]

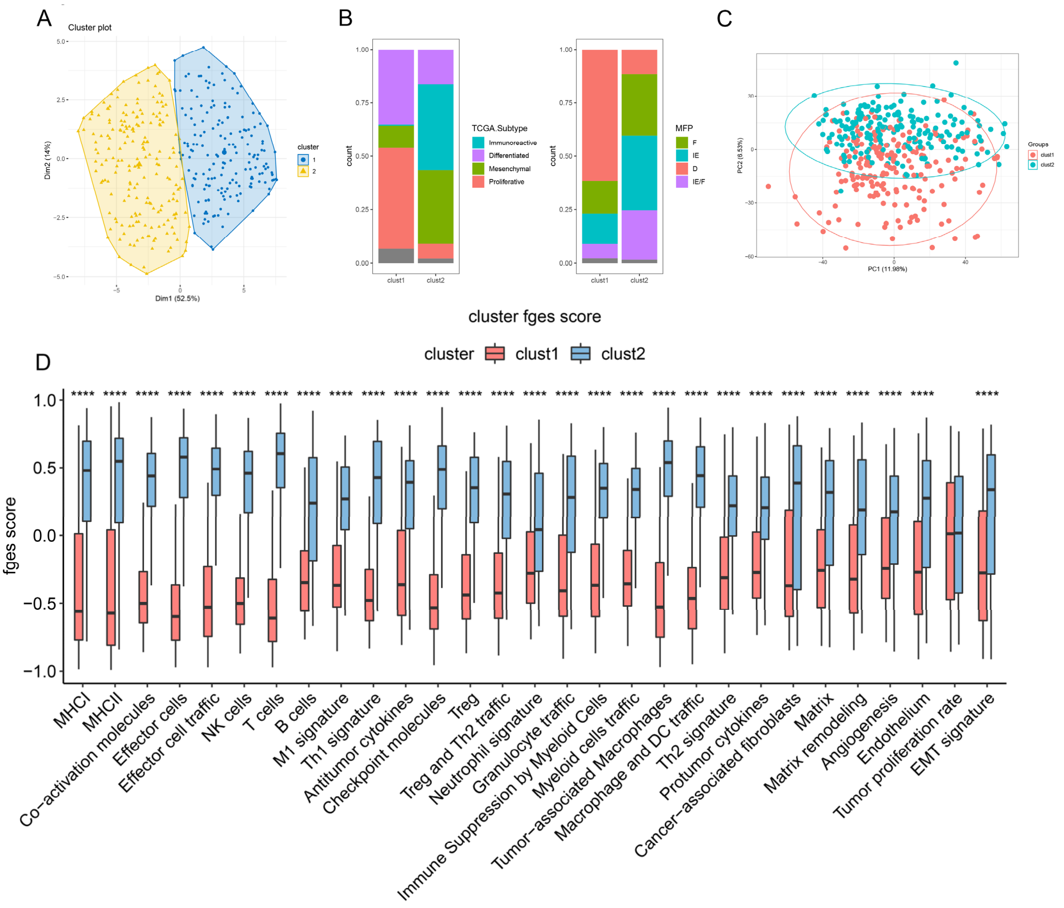

Supplementary Figure 1. Two ovarian cancer microenvironment subtypes were revealed by unsupervised analysis of the Fges. (A) Visualization of clustering results from K-means. (B) Percentages of OV subtypes identified by Bagaev et al. and TCGA Research Network across clust1 and clust2. (C-D) Top 10 pathways determined by KEGG (C) and GO (D) enrichment analysis of differential expression genes between clust1 and clust2. (E) Principal component analysis of transcriptome data. (F) Box plots showing differences in 17 immune MEs between clust1 and clust2.

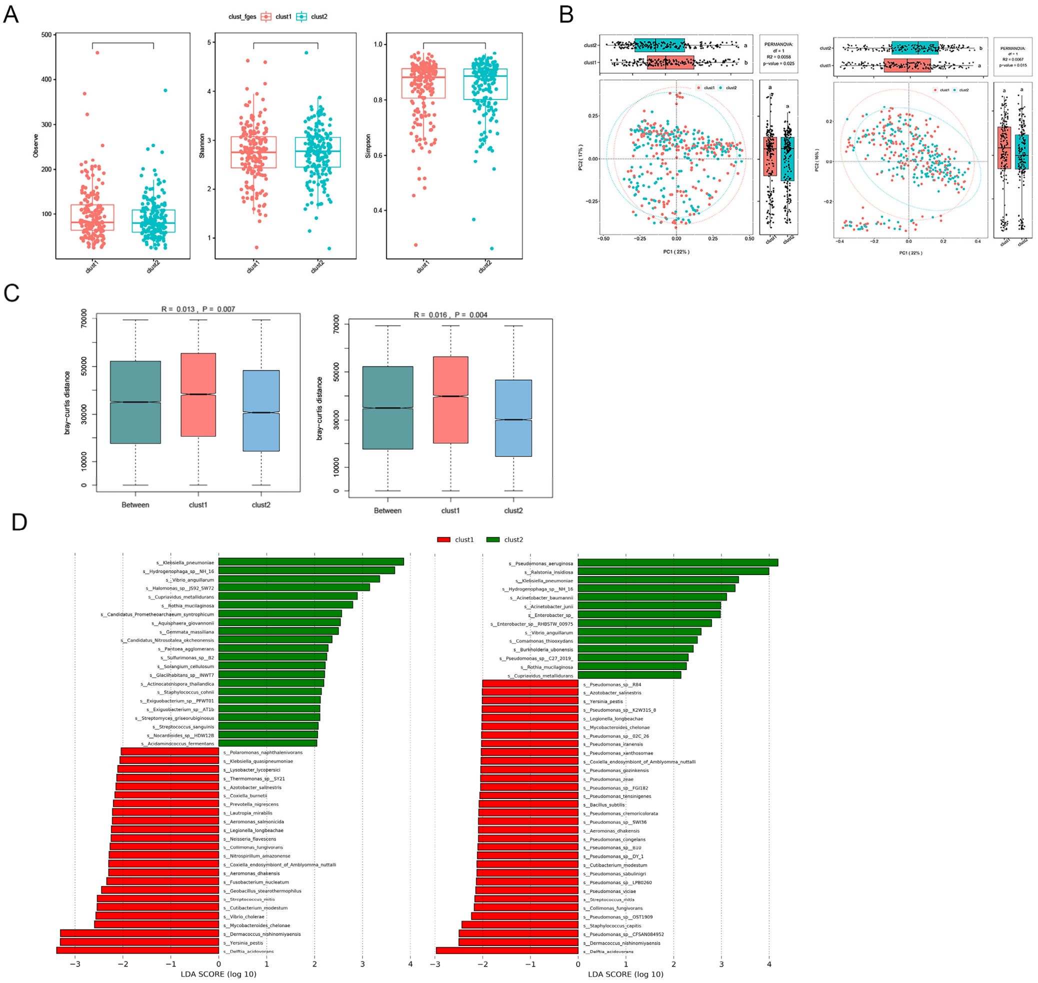

Supplementary Figure 2. (A) Boxplots of alpha-diversity compared between clust1 and clust2. (B) PCoA and boxplot on the Bray-cutis distances shown along the first two principal coordinates for strict-decontamination data (left) and raw microbiome composition data (right). (C) The Bray-Curtis distance of the microbial communities among individuals within the same group and between different groups for strict-decontamination data (left) and raw microbiome composition data (right). (D) Significant differentially abundant taxonomic biomarkers between clust1 and clust2 identified by LEfSe analysis for strict-decontamination data (left) and raw microbiome composition data (right).

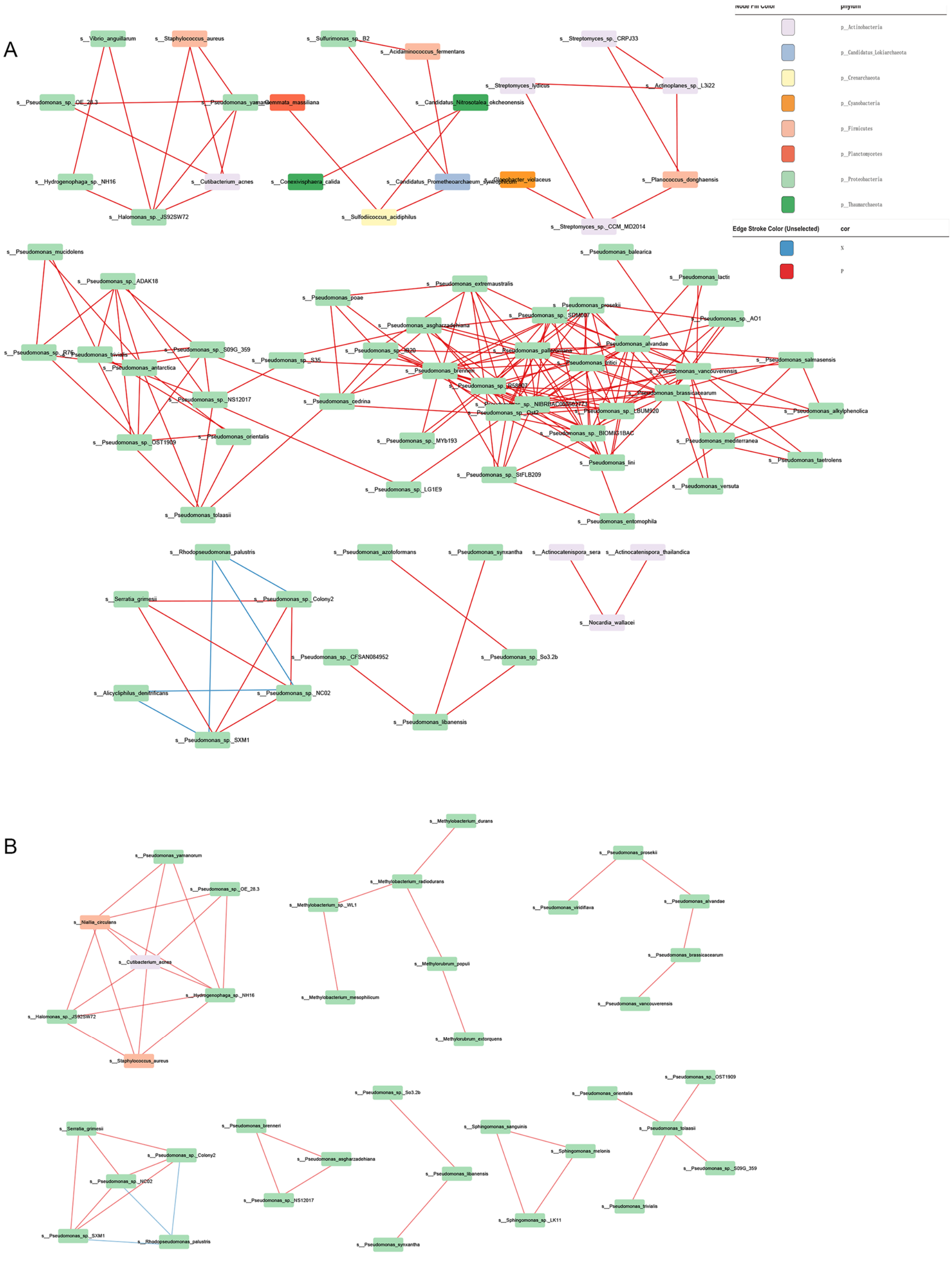

Supplementary Figure 3. Microbial co-abundance network of clust1(A) and clust2 (B). Connecting edges represent significant interactions and colored by correlation direction (red color for positive correlation, blue color for negative correlation).

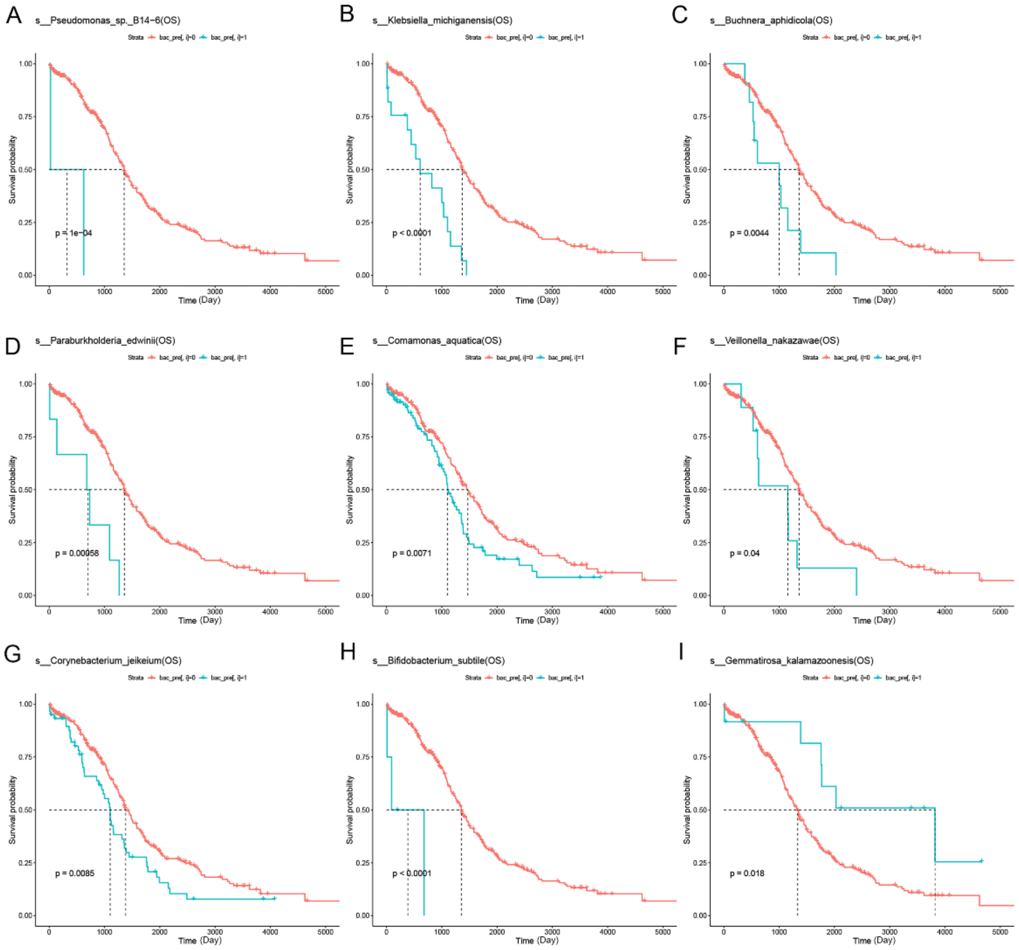

Supplementary Figure 4. Kaplan-Meier curves of DSS to the presence or not of *Pseudomonas sp. B14-6*(A); *Klebsiella michiganensis* (B); *Buchnera aphidicola* (C); *Paraburkholderia edwinii* (D); *Comamonas aquatica* (E); *Veillonella nakazawae* (F); *Corynebacterium jeikeium* (G); *Bifidobacterium subtile* (H) and *Gemmatirosa kalamazoonesis* (I).

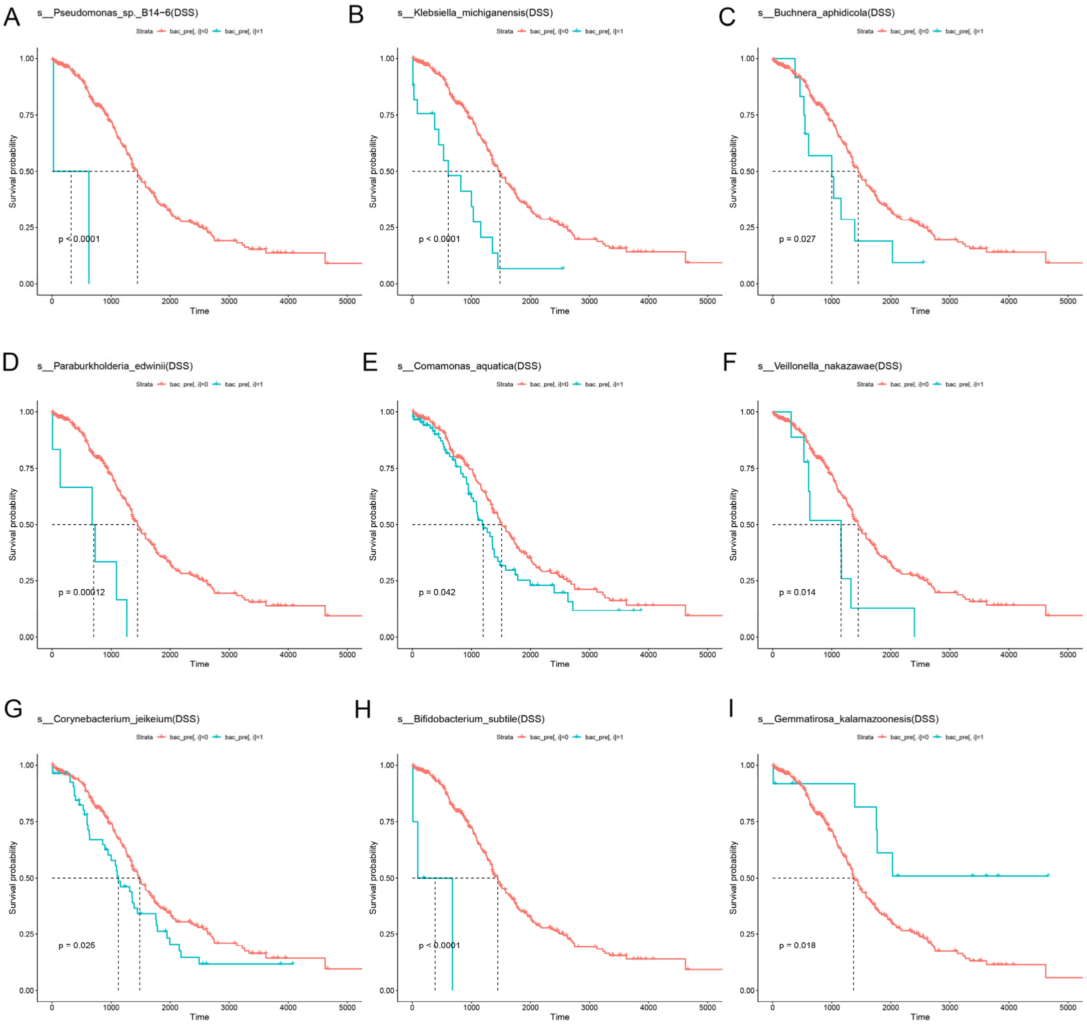

Supplementary Figure 5. Kaplan-Meier curves of DSS to the presence or not of *Pseudomonas sp. B14-6* (A), *Klebsiella michiganensis* (B), *Buchnera aphidicola* (C), *Paraburkholderia edwinii* (D), *Comamonas aquatica* (E), *Veillonella nakazawae* (F), *Corynebacterium jeikeium* (G) and *Bifidobacterium subtile* (H) and *Gemmatirosa kalamazoonesis* (I).

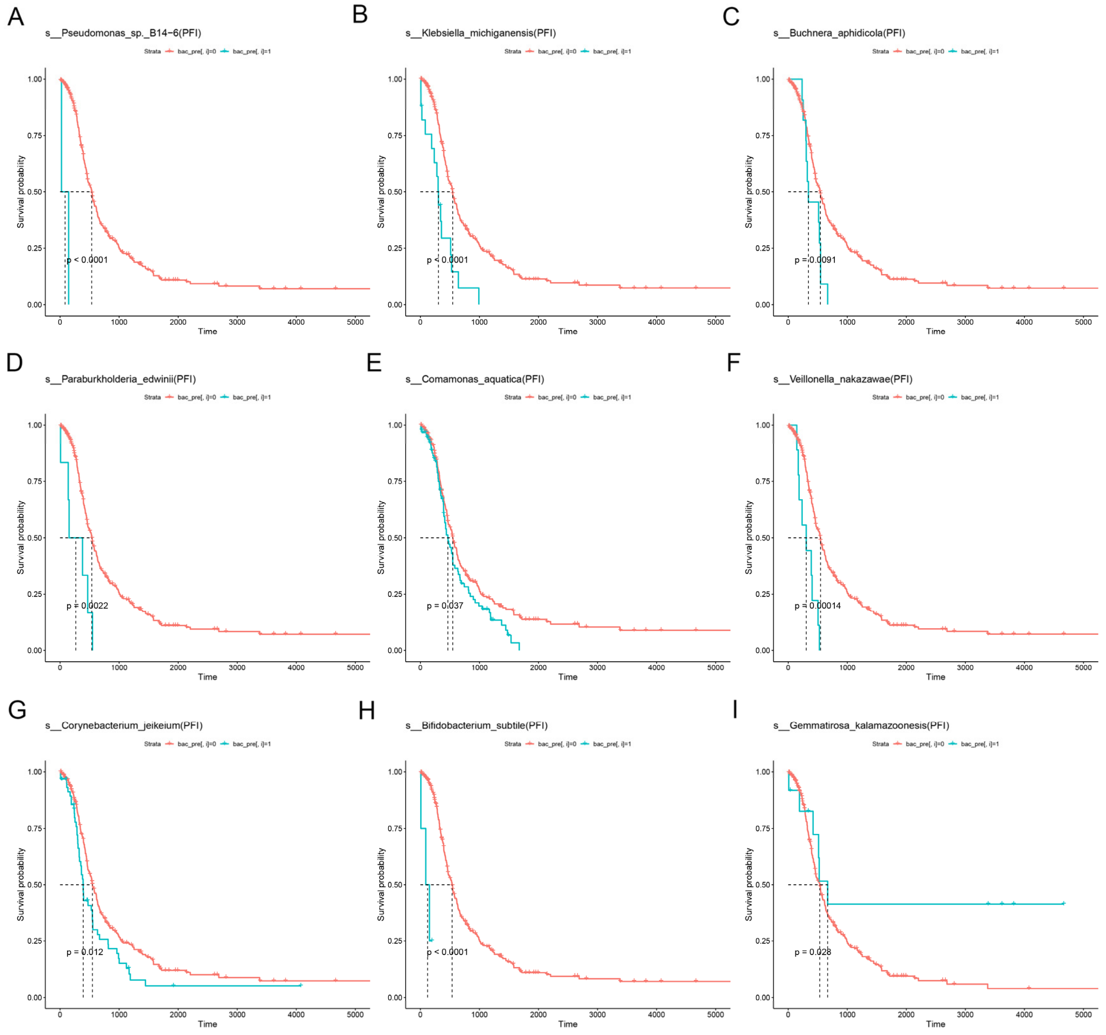

Supplementary Figure 6. Kaplan-Meier curves of PFI to the presence or not of *Pseudomonas sp. B14-6* (A), *Klebsiella michiganensis* (B), *Buchnera aphidicola* (C), *Paraburkholderia edwinii* (D), *Comamonas aquatica* (E), *Veillonella nakazawae* (F), *Corynebacterium jeikeium* (G) and *Bifidobacterium subtile* (H) and *Gemmatirosa kalamazoonesis* (I).

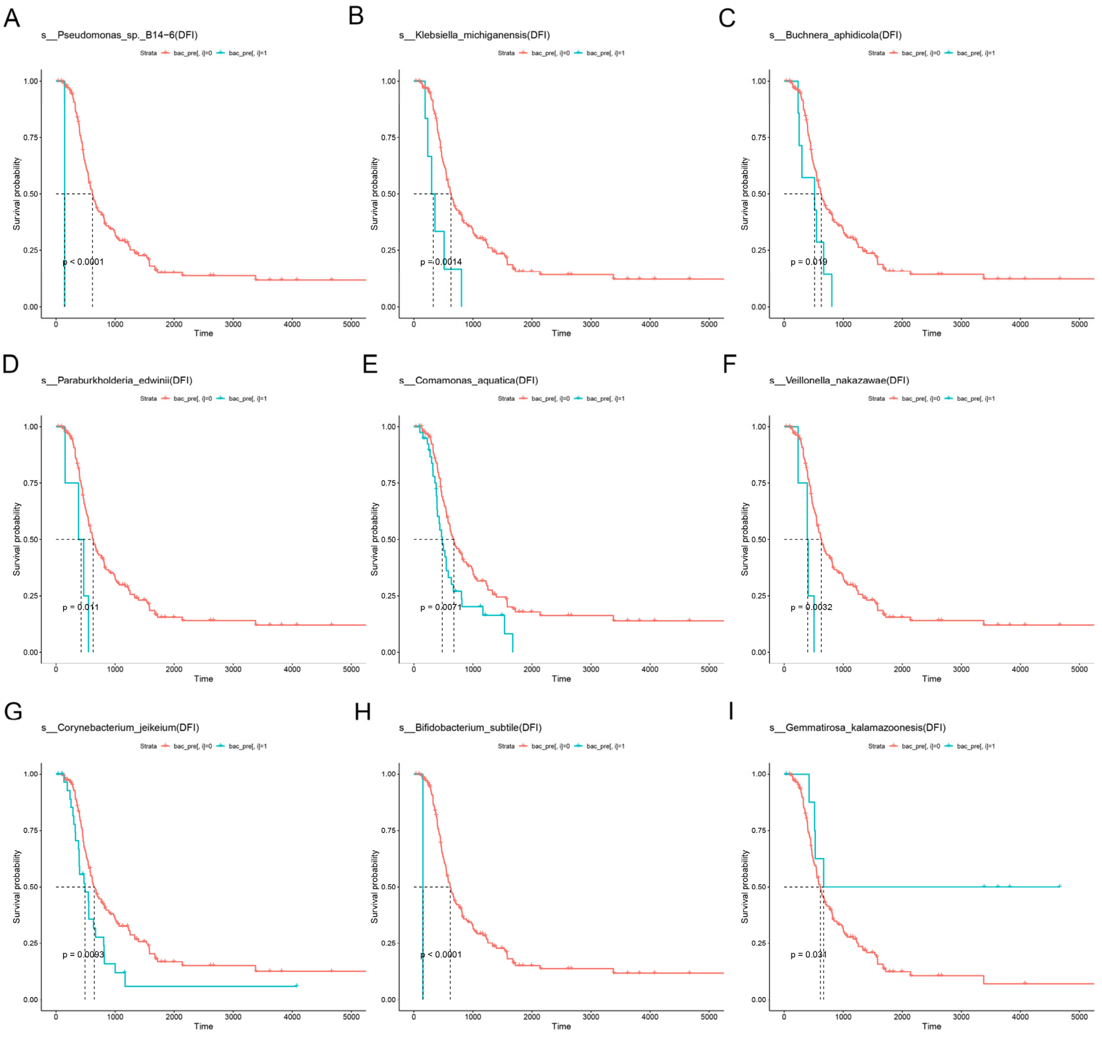

Supplementary Figure 7. Kaplan-Meier curves of DFI to the presence or not of *Pseudomonas sp. B14-6* (A), *Klebsiella michiganensis* (B), *Buchnera aphidicola* (C), *Paraburkholderia edwinii* (D), *Comamonas aquatica* (E), *Veillonella nakazawae* (F), *Corynebacterium jeikeium* (G) and *Bifidobacterium subtile* (H) and *Gemmatirosa kalamazoonesis* (I).

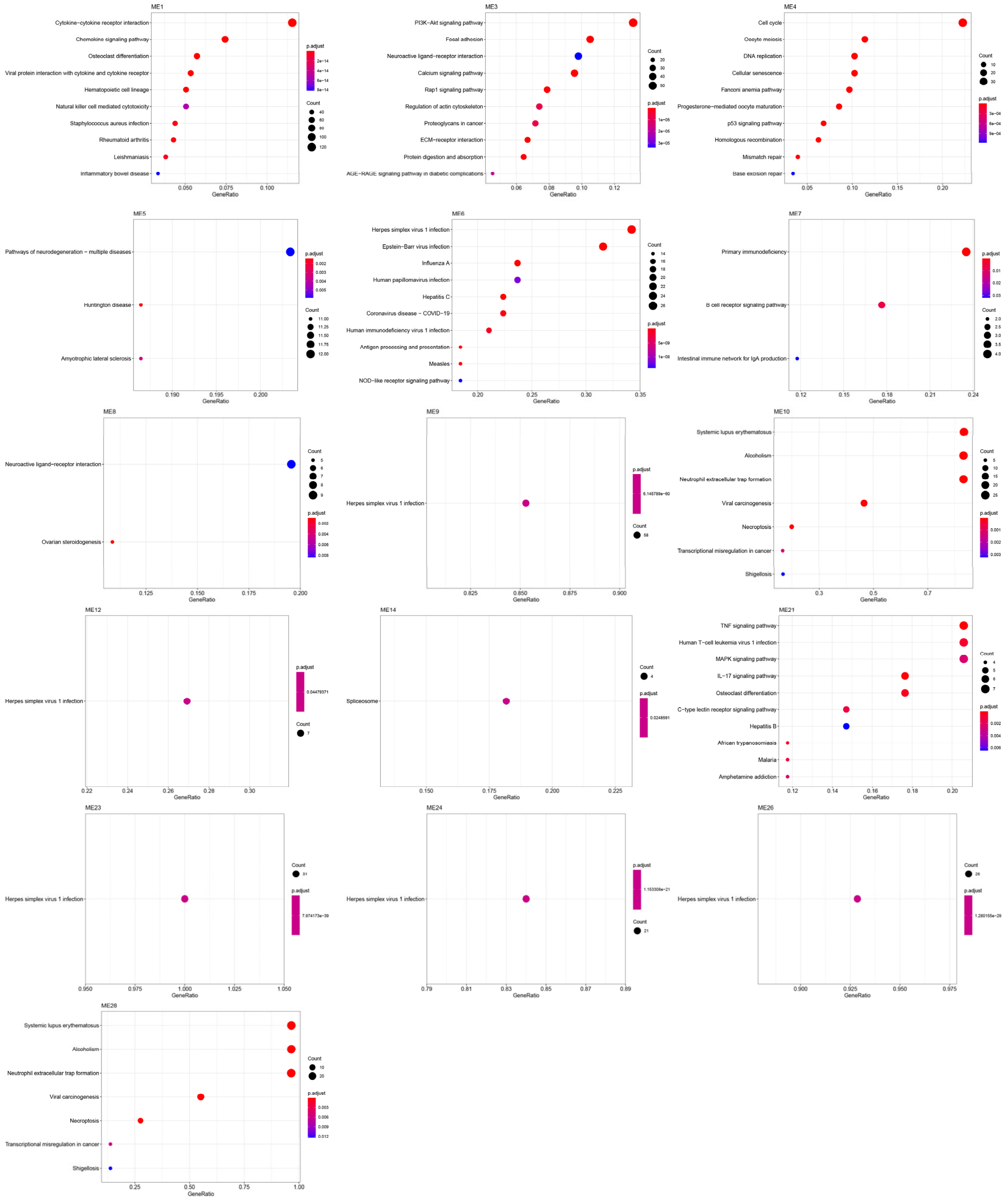

Supplementary Figure 8. Top 10 pathways determined by KEGG enrichment analysis of immune MEs.
